# Inhibitors of BMP‐1/tolloid‐like proteinases: efficacy, selectivity and cellular toxicity

**DOI:** 10.1002/2211-5463.12540

**Published:** 2018-11-12

**Authors:** Maya Talantikite, Pascaline Lécorché, Fabrice Beau, Odile Damour, Christoph Becker‐Pauly, Wen‐Bin Ho, Vincent Dive, Sandrine Vadon‐Le Goff, Catherine Moali

**Affiliations:** ^1^ Tissue Biology and Therapeutic Engineering Unit (LBTI) UMR5305, CNRS Univ Lyon Université Claude Bernard Lyon1 France; ^2^ CEA Saclay Institut Frédéric Joliot Direction de la recherche fondamentale SIMOPRO Gif‐sur‐Yvette France; ^3^ Banque de Tissus et Cellules Hospices Civils de Lyon France; ^4^ Institute of Biochemistry Unit for Degradomics of the Protease Web Christian‐Albrechts‐University Kiel Germany; ^5^ FibroGen Inc San Francisco CA USA

**Keywords:** astacin, cornea, extracellular matrix, fibrotic disorders, metalloproteinase, protease inhibition

## Abstract

BMP‐1/tolloid‐like proteinases belong to the astacin family of human metalloproteinases, together with meprins and ovastacin. They represent promising targets to treat or prevent a wide range of diseases such as fibrotic disorders or cancer. However, the study of their pathophysiological roles is still impaired by the lack of well‐characterized inhibitors and the questions that remain regarding their selectivity and *in vivo* efficiency. As a first step towards the identification of suitable tools to be used in functional studies, we have undertaken a systematic comparison of seven molecules known to affect the proteolytic activity of human astacins including three hydroxamates (FG‐2575, UK383,367, S33A), the protein sizzled, a new phosphinic inhibitor (RXP‐1001) and broad‐spectrum protease inhibitors (GM6001, actinonin). Their efficacy *in vitro*, their cellular toxicity and efficacy in cell cultures were thoroughly characterized. We found that these molecules display very different potency and selectivity profiles, with hydroxamate FG‐2575 and the protein sizzled being very powerful and selective inhibitors of BMP‐1, whereas phosphinic peptide RXP‐1001 behaves as a broad‐spectrum inhibitor of astacins. Their use should therefore be carefully considered in agreement with the aim of the study to avoid result misinterpretation.

**Abbreviations**
bFGF: basic fibroblast growth factor
BMP‐1: bone morphogenetic protein‐1
BTP: BMP‐1/tolloid‐like proteinase
DMEM: Dulbecco's modified Eagle's medium
ECM: extracellular matrix
HRMS: high‐resolution mass spectrometry
MTT: 3‐(4,5‐dimethylthiazol‐2‐yl)‐2,5‐diphenyltetrazolium bromide

Bone morphogenetic protein‐1 (BMP‐1) belongs to a small family of zinc‐dependent metalloproteinases, known as the BMP‐1/tolloid‐like proteinases (BTPs), which also includes in humans mammalian tolloid (mTLD, a splice variant of the *Bmp1* gene) and two homologous proteins, mammalian tolloid‐like 1 and 2 (mTLL‐1, mTLL‐2) 1. BTPs are composed of an astacin‐like catalytic domain followed by several complement C1r/C1s, Uegf, BMP‐1 (CUB) and epidermal growth factor (EGF) domains. They are members of the astacin metalloproteinase subgroup, which also comprises meprin α, meprin β and ovastacin 2. BMP‐1 (also known as procollagen C‐proteinase) and related tolloid proteinases were primarily identified as the main enzymes responsible for collagen maturation by excising the C‐propeptide of procollagens I‐III 3. Today, around 30 substrates have been described for BTPs 1, turning them into key players in regulating processes such as morphogenesis, tissue repair or tumour progression. These substrates include several fibrillar procollagens (types I, II, III, V, XI), small leucine‐rich proteoglycans (decorin, biglycan, osteoglycin), basement membrane components (laminin 332, procollagen VII, perlecan), lysyl oxidases (lysyl oxidase, lysyl oxidase‐like) and mineralization factors (dentin matrix protein‐1, dentin sialophosphoprotein). BTPs also activate numerous growth factors of the TGF‐β superfamily, either by direct cleavage of their prodomain (GDF‐8, GDF‐11), or by processing of antagonist molecules (LTBP, betaglycan or CD109 for TGF‐β1, chordin for BMP‐2/4, etc.) 1. Importantly, all BTPs seem to have the potential to cleave the same substrates, but their tissue distribution and catalytic efficiencies can differ significantly 4, 5. The catalytic domains of BTPs are very similar 6, and previous studies have shown that comparable results were found when testing inhibitors on several isoforms 7, 8. As the most active and widely distributed isoform, BMP‐1 was therefore used in the present study to represent the BTP family.

Fibrotic disorders are forms of defective tissue repair characterized by increased cell proliferation and deposition of extracellular matrix (ECM), particularly fibrillar collagens. They can affect numerous organs such as heart (cardiac fibrosis), liver (liver fibrosis further evolving to cirrhosis), kidneys (renal fibrosis), skin (hypertrophic scars, keloids) and cornea (corneal scarring) and are critical factors in progressive organ dysfunction, altogether contributing to 45% of all‐cause mortality worldwide 9. Through the maturation of ECM components and the activation of growth factors, BTPs play major roles during tissue remodelling. As such, they are recognized as attractive therapeutic targets to prevent or treat various fibrotic pathologies 10, 11, 12.

Inhibitors of zinc metalloproteinases are classically designed as small molecules bearing zinc‐binding groups which bind in the catalytic site and behave as competitive inhibitors. Following this strategy, a number of synthetic inhibitors of BTPs have been developed, most of them being hydroxamates 10, 13, 14, 15, 16. Some have shown promising activity in cell‐based 15, 17 or animal 18 models of fibrosis; however, none of them have entered the clinic. Hydroxamates can actually suffer from a set of drawbacks linked to *in vivo* instability and poor specificity due to their high affinity for metal ions 19. Other available inhibitors of BTPs include the *Xenopus* protein sizzled, which was previously found to be very potent and selective 7, and a newly developed phosphinic peptide inhibitor which is a broad‐spectrum compound targeting both BTPs and meprins. Phosphinic peptide inhibitors have the advantage of showing remarkable *in vivo* stability and have been successfully used in various animal models to selectively block their targets 20, 21.

Though some of these molecules have been designed to spare the activity of some important matrix metalloproteinases (MMPs), their inhibitory activity on other astacins is unknown. This specificity issue is crucial since it has been reported that BTPs and meprins have in common a striking specificity for acidic amino acids in the P_1_’ position (first residue C‐terminal to the scissile bond) 22 and that they share a number of common substrates and a high degree of conservation in their catalytic domains 23. Importantly, it was previously shown that, similarly to BTPs, meprin α and meprin β are able to perform the C‐terminal maturation of procollagens I and III 24, 25, making them further potential targets in collagen‐related diseases. However, meprins also have distinct functions during various (patho)physiological conditions and have been implicated in inflammation, cancer progression or Alzheimer's disease 23.

Here, we provide the first systematic comparison of three hydroxamates previously reported as powerful inhibitors of BMP‐1 10, 13, 16, 26, the newly developed phosphinic peptide and sizzled. The broad‐spectrum metalloprotease inhibitor GM6001 (also known as galardin or ilomastat), and actinonin, a naturally occurring hydroxamate and effective meprin inhibitor, were also included in the study. We have characterized their cellular toxicity and inhibitory efficacy, both *in vitro* and in cultures of HT1080 cells and human primary corneal fibroblasts, and have determined their selectivity towards BTPs and related meprins. Altogether, this study will be very useful to guide the choice of suitable tools for the inhibition of BTPs in future studies.

## Materials and methods

### Proteinases

Recombinant Flag‐tagged human BMP‐1 (http://www.chem.qmul.ac.uk/iubmb/enzyme/EC3/4/24/19.html) was produced in 293‐EBNA cells and purified as previously described 27. Meprin α (http://www.chem.qmul.ac.uk/iubmb/enzyme/EC3/4/24/18.html) and meprin β (http://www.chem.qmul.ac.uk/iubmb/enzyme/EC3/4/24/63.html) were produced in insect cells, purified and activated according to the protocols described in 28, 29.

### Inhibitors (Table 1)

Recombinant His‐tagged sizzled (from *Xenopus laevis*) was produced in 293‐EBNA cells and purified as described previously 7 (purity > 95% (SDS/PAGE), ESI‐MS m/z calcd 35481.8 found 35483.2). S33A was synthesized as described in 13 (purity > 97% (HPLC); HRMS (high‐resolution mass spectrometry; ESI (electrospray ionisation)) m/z: [MH+] calcd for C_27_H_33_N_4_O_6_S 541.2120; found 541.2119). FG‐2575 was designed and prepared by FibroGen, Inc. (purity > 95% (HPLC); MW = 503.6251; HRMS (ESI) m/z: [MH+] within 1.6 ppm of calcd value). The synthesis of the phosphinic peptide RXP‐1001 will be described elsewhere (Lecorché *et al*., in preparation; purity > 95% (HPLC); MS (matrix‐assisted laser desorption ionization) m/z: [MH+] calcd for C_55_H_67_N_8_O_14_P 1095.46; found 1095.57). UK383,367 and actinonin were purchased from Sigma‐Aldrich (L'isle d'abeau, France) and GM6001 from Enzo Life Sciences (Villeurbanne, France). All commercial compounds have purity > 98% (HPLC or TLC). Stock solutions of the inhibitors were prepared at 10 mm in DMSO, except for RXP‐1001 (3 mm in EtOH 50%) and sizzled (120 μm in HEPES 20 mm NaCl 0.5M pH 7.4).

**Table 1:** Protease inhibitors profiled in this study

Name	Type	Target	Formula
FG‐2575	Hydroxamate	BMP‐1	Undisclosed formula
UK383,367	Hydroxamate	BMP‐1	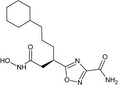
S33A	Hydroxamate	BMP‐1	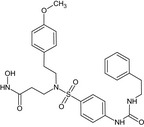
Sizzled	Xenopus protein	BMP‐1	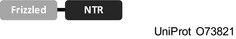
RXP‐1001	Phosphinic peptide	BMP‐1	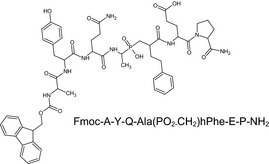
GM6001	Hydroxamate	MMP	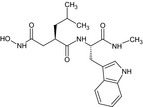
Actinonin	Hydroxamate	Meprin α	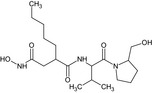

### Cell culture

Human primary keratocytes were obtained from human corneas (collected from three donors aged around 70), harvested at the ‘Banque de Tissus et Cellules’ of the ‘Hospices Civils de Lyon’ in agreement with French ethical regulations. Informed consent was obtained from the donors’ families, and the study conformed to the standards set by the Declaration of Helsinki. Keratocytes were grown in a medium without ascorbate shown to be optimal 30 to maintain their proliferation capacity and prevent their differentiation into myofibroblasts [Dulbecco's modified Eagle's medium (DMEM)/HamF12 1 : 1 (GE Healthcare, Velizy, France), 10% iron‐supplemented newborn bovine serum (Hyclone, Fisher scientific, Illkirch, France), 1% antibiotic/antimycotic solution (AAS, Sigma‐Aldrich) and 5 ng·mL^−1^ basic fibroblast growth factor (bFGF) (Sigma‐Aldrich)]. These culture conditions enabled easy detection of both unprocessed procollagen I and released C‐propeptide. Cells were used between passage 6 and passage 9.

HT1080 cells (ATCC CCL‐121) were grown in DMEM with 10% foetal bovine serum (FBS) (GE Healthcare) and 1% AAS.

Bone morphogenetic protein‐1‐overexpressing HT1080 cells were obtained as previously described 31 and grown in DMEM with 10% FBS and 500 μg·mL^−1^ G418 sulphate (GE Healthcare). For inhibitor evaluation, 20 000 cells·well^−1^ (in 12‐well plates) were treated with 2.5 μm of synthetic inhibitors, 0.15 μm of sizzled or with an equal volume of vehicle starting 4 h after seeding. After 2 days, confluent cells were washed with PBS and maintained in serum‐free medium (supplemented in inhibitor) for another 24–48 h. Modification of the cell morphology was visualized on a Nikon Eclipse TE300 Inverted Microscope.

### Inhibition assay

To determine inhibitor potencies, the fluorogenic peptide substrate Mca‐Y‐V‐A‐D‐A‐P‐K‐Dnp (20 μm; Enzo Life Sciences), which can be cleaved by both BTPs and meprins 7, was used as a substrate (*K*_m_ = 7.2, 15 and 9 μm for BMP‐1, meprin α and meprin β, respectively). It was incubated with 12 nm BMP‐1, 1 nm meprin α or 0.5 nm meprin β, in the presence of increasing concentrations of the inhibitors (0.01–100 μm) in 50 mm HEPES pH 7.4, 0.15 m NaCl, 5 mm CaCl_2_, 0.02% octyl β‐D‐glucopyranoside. RXP‐1001 was also assayed at pH 6.8. The cleavage assays were performed in 96‐well nonbinding, flat‐bottomed black plates (Corning, Amsterdam, The Netherlands) in 100 μL total reaction volume at 37 °C, and linear increase in fluorescence was monitored during 20 min using an Infinite M1000 Tecan reader (excitation wavelength: 320 nm; emission wavelength: 405 nm). IC_50_ values were determined by plotting the ratio of the inhibited versus uninhibited enzyme activities against the inhibitor concentration. Nonlinear regression analysis and calculations were performed using prism 5 (GraphPad Software, La Jolla, CA, USA) (*n* = 3).

### MTT Assay

HT1080 cells and keratocytes were seeded into 96‐well culture plates (Corning) at a density of 1500 and 4000 cells·well^−1^, respectively. After 48 h, cells were treated with various concentrations of the inhibitors, or with the corresponding vehicle, in serum‐free medium (with AAS and bFGF for keratocytes), for 24 or 48 h. 3‐(4,5‐dimethylthiazol‐2‐yl)‐2,5‐diphenyltetrazolium bromide (MTT) reagent (Sigma‐Aldrich) was then added to each well and incubated with the cells at 37 °C for 3 h. The supernatant was removed and a DMSO:ethanol (1/1) solution added to each well to dissolve the formazan crystals. Absorbance was measured at 570 nm using the Infinite reader. 100% values were obtained with cells treated with vehicle only.

### Procollagen cleavage assay

Keratocytes were seeded in 24‐well plates (Corning, 20 000 cells·well^−1^) and grown for 2 days (until confluence reached 90%). They were then washed three times with PBS followed by addition of serum‐free, phenol red‐free DMEM supplemented with AAS and bFGF and containing different concentrations of the inhibitors. After 48 h, the conditioned medium of each well was collected, clarified by centrifugation and boiled 5 min in SDS/PAGE sample buffer containing DTT. Equal volumes of each sample were loaded on 4–20% polyacrylamide gradient gels (Bio‐Rad, Marnes La Coquette, France), followed by transfer to PVDF membranes (Millipore, St Quentin en Yvelines, France) and analysis by western blotting using the LF‐41 rabbit antibody directed against the procollagen I C‐propeptide (kind gift of L. Fisher, NIH Bethesda, USA) as primary antibody and goat HRP‐linked anti‐rabbit antibody (Cell Signaling, Leiden, The Netherlands) as secondary antibody. Protein bands were detected using the Clarity western solution (Bio‐Rad) and a Fusion camera (Vilber‐Lourmat, Marne‐la‐Vallée, France) then quantified by densitometry using ImageQuant TL (GE Healthcare).

## Results

The molecules selected for this study are as follows (Table 1): hydroxamates FG‐2575 (sometimes referred to as BI‐1 8, 26), UK383,367 16 and S33A 13, the sizzled protein 7 and the recently developed phosphinic peptide RXP‐1001. GM6001 and actinonin, whose activity towards BTPs had never been documented, were also included.

### *In vitro* efficacy

The inhibitors were first profiled at pH 7.4 for their ability to block the cleavage of a fluorogenic peptide substrate by the three major human astacins (BMP‐1, meprin α and meprin β) 7 and ranked according to their half‐maximal inhibitory concentration (IC_50_, Table 2). For BMP‐1, these potencies can be ordered as follows: FG‐2575 > S33A ≈ sizzled > UK383,367 > RXP‐1001 >> actinonin > GM6001. Thus, the results clearly show that, in these conditions, FG‐2575 is the most powerful inhibitor of BMP‐1 (IC_50_ = 2.9 ± 0.3 nm). Together with the *Xenopus* protein sizzled (IC_50_ = 8.5 ± 0.5 nm), it also appears to be highly specific for BMP‐1 as their IC_50_ values for meprins are more than 1000‐fold higher. With 5‐ to 20‐fold higher IC_50_s, the other hydroxamates remain good inhibitors of BMP‐1, but do not demonstrate the same level of specificity as they also inhibit meprin α with an IC_50_ around 300 nm. As it was previously demonstrated that inhibition by phosphinic peptides, in contrast to hydroxamates, involves a proton uptake by a glutamate which is conserved in all metzincins 32, 33, RXP‐1001 was also tested at pH 6.8. This leads to significantly increased potencies for all 3 enzymes (by a factor between 2.7 and 6.9). Interestingly, RXP‐1001 was also the only molecule that showed a high degree of potency against all three enzymes (around micromolar or below at the two tested pH), and thus, it can be viewed as a broad‐spectrum inhibitor of human astacins. GM6001 and actinonin were inactive on BMP‐1 (IC_50_ > 10 μm), but both appeared as good inhibitors of meprin α, as already reported 34. Actinonin also displayed some inhibitory activity towards meprin β.

**Table 2:** Half‐maximal inhibitory concentrations of the inhibitors for meprin α, meprin β and BMP‐1 at pH 7.4 or (*)6.8. A representative example of data determination (FG‐2575 on BMP‐1) is given in the Fig. S1

Compound	IC_50_ (nm)^a^		
	Meprin α	Meprin β	BMP‐1
FG‐2575	> 1000	> 10 000	2.9 ± 0.3
UK383,367	249 ± 17	> 3000	55 ± 15
S33A	393 ± 82	> 10 000	7.9 ± 0.6
Sizzled	> 1000	> 10 000	8.5 ± 0.3
RXP‐1001	245 ± 40 (42 ± 9*)	1300 ± 100 (484 ± 33*)	359 ± 20 (52 ± 3*)
GM6001	185 ± 12	> 10 000	> 100 000
Actinonin	55 ± 11	744 ± 29	> 10 000

a Mean values ± SD of three independent experiments performed in duplicate.

### Cellular toxicity of the inhibitors

**Figure 1:**
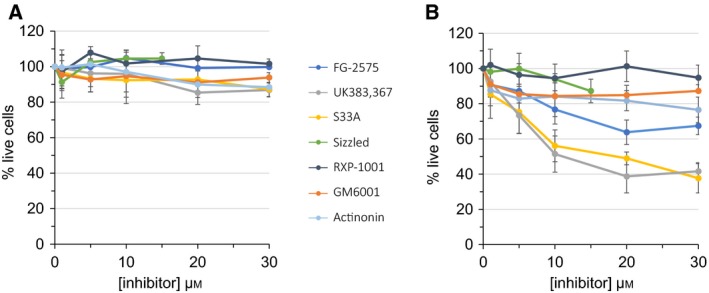
Evaluation of the cellular toxicity of the inhibitors. Viability of human keratocytes (A) or HT1080 cells (B) treated with increasing concentrations of inhibitors was measured using the MTT assay. Cells were seeded and grown for 2 days, washed with PBS and treated with inhibitors in serum‐free medium for 48 (keratocytes) or 24 (HT1080) h. 100%: vehicle only. Mean ± SD,* n* = 3.

In order to carefully choose the concentrations to be used in cell‐based assays, evaluation of the toxicity of the inhibitors was needed. Viability of human primary keratocytes (corneal stromal cells) and HT1080 cells (fibrosarcoma‐derived cancer cells) treated with increasing concentrations of the seven compounds was assessed using the MTT viability assay in serum‐free conditions. The two cell types are very different in terms of origin, metabolism and nature of cell–matrix interactions (e.g. no fibrillar collagens are secreted by HT1080 cells) and, as can be seen in Fig. 1, responded differently to the addition of the inhibitors. Keratocytes were found to be very resistant, and even when 30 μm of the compounds was added to the cells, their viability was not significantly reduced (Fig. 1A). HT1080 cells were more sensitive to the cytotoxic effects of the molecules (Fig. 1B), at least in the serum‐free conditions of the assay. For this reason, viability of HT1080 was measured after 24 h only, and DMSO concentration was kept below 0.3%, since it started to show some toxicity at around 1%. Whereas actinonin and GM6001 were essentially harmless to HT1080 cells, UK383,367 and S33A were strongly cytotoxic at concentrations above 10 μm. FG‐2575 was better tolerated, with a 30% drop in viability above 20 μm, which is a concentration 4 orders of magnitude above its active concentration. RXP‐1001 was the least toxic compound since even at 30 μm it did not significantly modify HT1080 viability. Sizzled could not be evaluated at concentrations above 15 μm, but even at this relatively high concentration for a protein, it had little influence on HT1080 viability.

### Effect on BMP‐1 activity in human keratocytes

**Figure 2:**
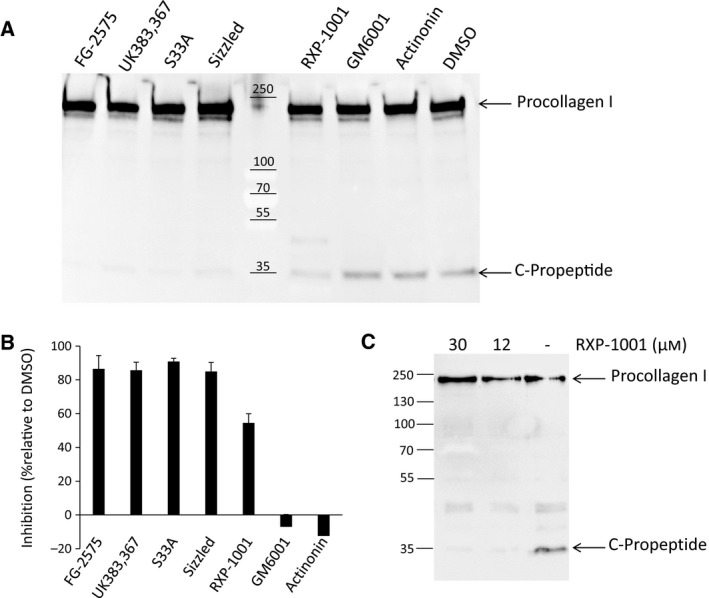
Effects of BTP inhibitors on the BMP‐1‐dependent maturation of procollagen I. (A–C) Subconfluent human keratocytes were treated with inhibitors or vehicle for 48 h. Medium was harvested, assayed by western blotting and probed with an antibody raised against the C‐terminal propeptide of procollagen type I. (A) Treatment with 2.5 μm of inhibitors except sizzled which was assayed at 0.25 μm. (B) Quantification of band intensities in A. (C) Assay of RXP‐1001 at two higher concentrations. One representative experiment out of 3 independent experiments is shown in all panels.

To evaluate the inhibitors in a primary cell culture assay, we selected human keratocytes which were previously found to represent a good cellular system to study substrate processing by physiological levels of BMP‐1 31 and which are the main cell type involved in corneal scarring, a condition where BMP‐1 is of therapeutic interest 12. Adult keratocytes are also known to deposit large amounts of collagen I, and they were used in our study to monitor the effect of the selected molecules on the BMP‐1‐dependent maturation of procollagen I into pN‐collagen and C‐propeptides. Subconfluent keratocytes were treated at the single concentration of 2.5 μm (synthetic inhibitors) or 0.25 μm (sizzled) for 48 h in serum‐free medium and compared to untreated cells. Media were harvested and assayed for procollagen processing using western blotting and an antibody raised against the C‐terminal propeptide of type I procollagen (Fig. 2A). In the absence of inhibitor or in the presence of actinonin or GM6001, C‐propeptide release was easily detected. In agreement with the *in vitro* data, other compounds were found to efficiently block this processing, as indicated by the decreased amounts of C‐propeptide seen in Fig. 2A. Despite the difference in initial concentrations, the same level of inhibition was obtained with sizzled and the hydroxamates (around 90%, as quantified by western blotting, Fig. 2B). In contrast, RXP‐1001, which efficiently inhibited recombinant BMP‐1*,* required a concentration of 12 μm to reach the same efficiency (Fig. 2C).

### Effect on HT1080 cells overexpressing BMP‐1

**Figure 3:**
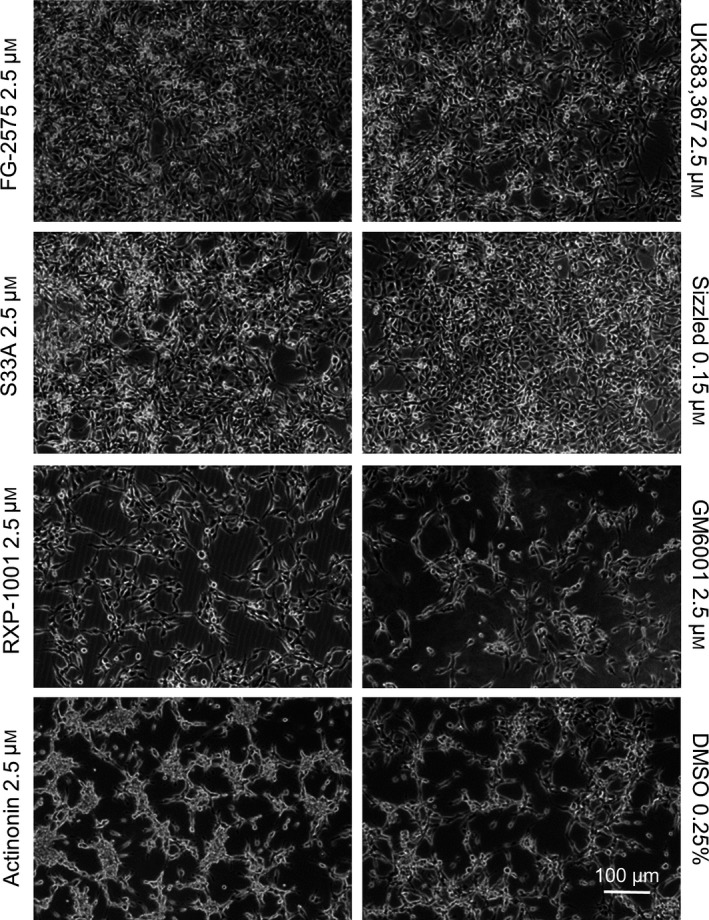
Effects of BTP inhibitors on BMP‐1‐induced HT1080 change of cell morphology: Phase‐contrast images of HT1080 cells stably overexpressing BMP‐1, grown in the presence of vehicle or inhibitors, 24 h after serum deprivation. Cells treated with BMP‐1 inhibitors at 2.5 μm (FG‐2575, UK383,367, S33A and sizzled) show normal HT1080 morphology. On the contrary, untreated cells (DMSO panel) or cells treated with molecules that do not inhibit BMP‐1 (GM6001, actinonin) exhibit changes in morphology and start to detach from culture plates, indicative of the presence of active BMP‐1. RXP‐1001 at 2.5 μm has an intermediate effect, with only partial reversal of the phenotype. The 0.25% DMSO condition is shown as a representative control of all vehicle conditions. Scale bar: 100 μm.

HT1080 cells do not express fibrillar collagens but were previously shown to secrete several established or putative BMP‐1 substrates 31. Also, when BMP‐1 is transfected in HT1080 cells, a striking phenotype is induced, with cells losing their rounded shape and starting to detach from the culture plate when reaching high density (Fig. 3, DMSO panel). This change in cell morphology is due to the catalytic activity of BMP‐1 since it is not present when a similar construct encoding an inactive BMP‐1 mutant (E94A, Anastasi *et al*., under revision) is used. We took advantage of this marked phenotype, which is a direct readout of BMP‐1 activity, to assess the activity of the selected molecules in another type of cell‐based assay.

As can be seen in Fig. 3, actinonin and GM6001, which do not inhibit BMP‐1 *in vitro*, had no impact on the phenotype, with cells behaving like untreated (DMSO) cells. In contrast, established inhibitors of BMP‐1 had a remarkable effect, leading to the reversal of the adhesion phenotype when cells were grown in the presence of these compounds. Similar to the results obtained with keratocyte cultures, sizzled was efficient at a concentration as low as 0.15 μm, with cells maintaining their normal aspect, while the hydroxamates and RXP‐1001 required higher concentrations. Phenotype reversal was observed for hydroxamates at 2.5 μm, whereas, like for keratocytes, RXP‐1001 was less efficient at this concentration.

## Discussion

This work was initiated in the course of our study on the role of BMP‐1 in corneal scarring 12, for which we needed to select a potent, selective and nontoxic BMP‐1 inhibitor. We wanted to be sure that only BTPs would be targeted by the selected molecule but realized that, except for sizzled, there was no information about the selectivity of BTP inhibitors towards meprins. This is of utmost importance since BTPs and meprins have both common and specific substrates and, even for common substrates, the cleavage pattern can be significantly different leading to distinct physiological effects 24, 25. Also, previous studies on other metalloproteases such as MMPs have demonstrated that the lack of selectivity of metalloproteinase inhibitors can lead to erroneous conclusions and to dramatic side effects in clinical trials 35. Noteworthy, FG‐2575, S33A, UK383,367, RXP‐1001 and sizzled have been tested on representative panels of MMPs and found to have no significant effects 7, 13, 16. In contrast, the natural antibiotic actinonin was previously shown to inhibit aminopeptidases or peptide deformylases 36 and GM6001 is a broad‐spectrum inhibitor of several metalloproteases 37.

The five established BTP inhibitors showed very different inhibition profiles. One major result of our study is that sizzled and FG‐2575 clearly stand out as the most potent and selective compounds with low nanomolar activities on BMP‐1 and more than 1000‐fold higher IC_50_s for meprins. Sizzled has previously been shown to efficiently inhibit the cleavage of other BMP‐1 substrates such as procollagen III, procollagen V and pro‐lysyl oxidase 7. FG‐2575 was also used to inhibit the cleavage of laminin‐332 both *in vitro* and in cultures of human keratinocytes and to prevent pro‐lysyl oxidase maturation in 293‐EBNA cells overexpressing the protein 8, 17. These two compounds actually behaved very well in our cell‐based assays combining efficiency and low cytotoxicity and appear to be the most attractive tools to modulate BTP activity both *in vitro* and *in cellulo*. BTPs being known to be important for matrix assembly, their inhibition could interfere with the deposition of the provisional matrix needed to support cell adhesion. It is worth noting that we did not observe any detrimental effect of the addition of the inhibitors in our culture conditions, 4 (HT‐1080) or 48 h (MTT assay) after seeding. However, further studies will be needed to obtain information about the usability and pharmacokinetic/biodistribution properties of sizzled and FG‐2575 in whole‐organism experiments.

In our cell‐based assays, we found that the inhibitory capacity of sizzled towards BMP‐1 is stronger than that of FG‐2575. However, this advantage might be counterbalanced by the increased production cost of sizzled compared to a small chemical compound, by the size of the protein (30 kDa) which may hamper its diffusion within target tissues and by the possible immunogenicity of a protein originating from *Xenopus*. These various parameters will have to be carefully evaluated in the future.

Though slightly less active on BMP‐1, RXP‐1001 is the only compound tested here to be a broad‐spectrum inhibitor of BTPs and meprins, which makes it a very valuable tool to explore redundancy and cross‐reactivity of astacins. In particular, as our previous results show that meprins can directly contribute to collagen deposition in tissues 24, 25, it could be very informative to evaluate the impact of blocking all procollagen C‐proteinase activities simultaneously in fibrosis models. However, RXP‐1001 seems somewhat less potent in cell cultures than other synthetic compounds. This can be compensated by the use of higher RXP‐1001 concentrations without any obvious toxicity for the two cell types tested here. This apparent loss of inhibitory potency may be explained by its relatively high pH sensitivity around physiological pH or the possible sequestration of RXP‐1001 by residual serum proteins present in the medium. As already observed for this type of compounds 38, this sequestration mechanism can be favoured by the hydrophobic residues and negative charges born by the phosphinic peptide. This property can be a real benefit *in vivo*, since it will hamper rapid clearance and help the transport of the molecule to pathological sites. Also, a previous report by Fish *et al*. 15 suggested that ionized BTP inhibitors may have a lower efficacy *in cellulo* compared to neutral compounds, possibly because of repulsive interactions with the biomolecules present in the specific microenvironments where BMP‐1 is located.

This study also shows that actinonin can be used to modulate meprin activity with no impact on BTPs, whereas S33A and UK383,367 target both BMP‐1 and meprin α, with little effect on meprin β. Finally, the widely used MMP inhibitor GM6001 does not affect BMP‐1 and meprin β in the tested concentration range but a clear off‐target activity of this molecule on meprin α is observed. This is an illustrative example of why careful comparison is necessary before choosing an inhibitor to probe enzymatic activity.

In summary, this study reveals striking differences in the potency and selectivity of the inhibitors tested. It demonstrates that some of the molecules sometimes claimed to be selective BMP‐1 inhibitors by commercial suppliers (UK383,367) actually also inhibit meprin α. Also, the marked differences in inhibition profiles suggest that the full spectrum of activities (from very selective of one family member to family‐wide) can be achieved and confirm that there are both common and specific features in the catalytic sites of BTPs and meprins, as previously shown by crystallography 6, 39. The study also highlights for the first time the advantages and drawbacks of a diverse range of inhibitors that can be picked as required to modulate either one, two or the three proteases. It should help to select the right tool for the right experiment and pave the way towards novel *in vivo* applications of BTP inhibitors, for which only few examples have been described to date 18.

## Author contributions

SVLG and CM designed and coordinated the study, MT and SVLG performed the experiments, SVLG, PR, FB, WBH, CBP and VD produced and purified the enzymes and/or inhibitors used in this study. OD provided the keratocytes. SVLG, MT and CM analysed and interpreted the data, and wrote the paper. All authors reviewed the results, critically revised the manuscript and approved the final version.

## Conflict of interest

WBH is an employee of FibroGen, Inc. The other authors report no conflict of interests.

## Supporting information


**Fig. S1**. Representative example of inhibition assay and IC_50_ determination. (A) Inhibition of BMP‐1 (12 nm) by increasing concentrations of FG‐2575 (0–100 nm, each concentration in duplicate). Linear increase in fluorescence was monitored during 20 min (substrate concentration 20 μm, excitation wavelength 320 nm, emission wavelength 405 nm). Enzyme activity is given by the slope of the curve, determined by linear regression using Excel. One representative experiment out of 3 independent experiments is shown. (B) IC_50_ determination of FG‐2575 on BMP‐1. Plot of % of residual activity (ratio of inhibited versus uninhibited enzyme activities) against FG‐2575 concentration. Mean ± SD of 3 independent experiments performed in duplicate. IC_50_ is determined by nonlinear regression using GraphPad Prism 5.
