# Supplementary figures and images for: Inhibitors of BMP‐1/tolloid‐like proteinases: efficacy, selectivity and cellular toxicity
Source: FEBS Open Bio. 2018 Nov 12;8(12):2011–21. doi: 10.1002/2211-5463.12540 (PMC6275283; doi:10.1002/2211-5463.12540)

Supplementary Figure

A

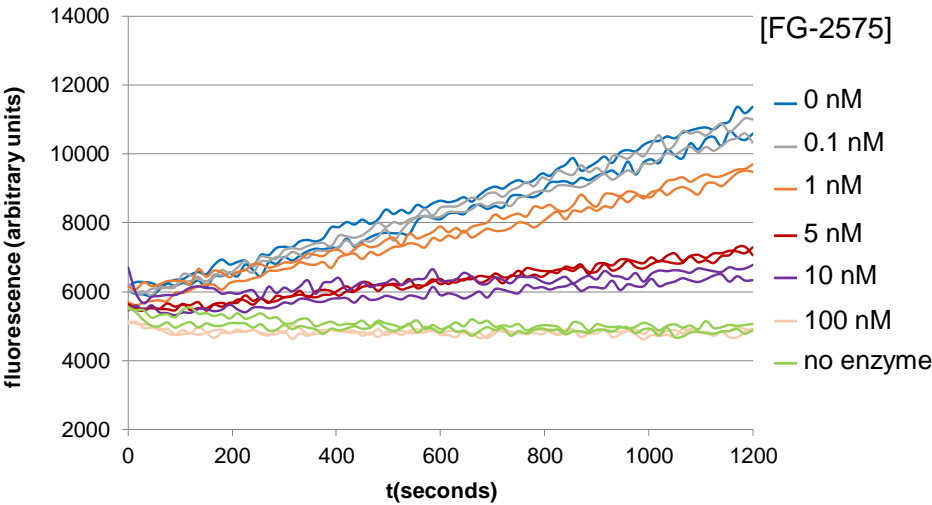

B

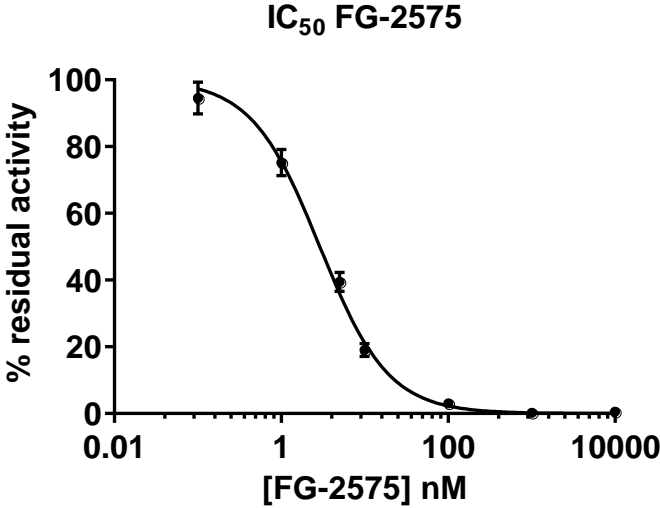

Supplement: Supplementary file 1 — Fig. S1. Representative example of inhibition assay and IC50 determination. (A) Inhibition of BMP‐1 (12 nm) by increasing concentrations of FG‐2575 (0–100 nm, each concentration in duplicate). Linear increase in fluorescence was monitored during 20 min (substrate concentration 20 μm, excitation wavelength 320 nm, emission wavelength 405 nm). Enzyme activity is given by the slope of the curve, determined by linear regression using Excel. One representative experiment out of 3 independent experiments is shown. (B) IC50 determination of FG‐2575 on BMP‐1. Plot of % of residual activity (ratio of inhibited versus uninhibited enzyme activities) against FG‐2575 concentration. Mean ± SD of 3 independent experiments performed in duplicate. IC50 is determined by nonlinear regression using GraphPad Prism 5. [file FEB4-8-2011-s001.pdf]
